# Neurological update: neuro-otology 2023

**DOI:** 10.1007/s00415-023-11922-9

**Published:** 2023-08-17

**Authors:** Gábor M. Halmágyi, Gülden Akdal, Miriam S. Welgampola, Chao Wang

**Affiliations:** 1 Neurology Department, Royal Prince Alfred Hospital, Sydney, Australia; 2 Central Clinical School, University of Sydney, Sydney, Australia; 3 Neurology Department, Dokuz Eylül University Hospital, Izmir, Turkey; 4 Neurosciences Department, Dokuz Eylül University Hospital, Izmir, Turkey

**Keywords:** Head impulse test, Positional vertigo, Meniere’s disease, Vestibular migraine, Vestibular event monitoring, Vestibular machine learning

## Abstract

Much has changed since our last review of recent advances in neuro-otology 7 years ago. Unfortunately there are still not many practising neuro-otologists, so that most patients with vestibular problems need, in the first instance, to be evaluated and treated by neurologists whose special expertise is not neuro-otology. The areas we consider here are mostly those that almost any neurologist should be able to start managing: acute spontaneous vertigo in the Emergency Room—is it vestibular neuritis or posterior circulation stroke; recurrent spontaneous vertigo in the office—is it vestibular migraine or Meniere's disease and the most common vestibular problem of all—benign positional vertigo. Finally we consider the future: long-term vestibular monitoring and the impact of machine learning on vestibular diagnosis.

## The patient with recurrent acute vertigo attacks

Amongst patients seen by appointment, complaining of what after careful interrogation sounds like recurrent acute vertigo attacks, the differential diagnosis is basically limited to benign positional vertigo (BPV), Meniere’s disease (MD) or vestibular migraine (VM) [1]. Patients who start to have posterior circulation transient ischemic attacks with predominant vertigo will usually have a stroke long before their appointment comes around [2].

## Benign positional vertigo (BPV)

BPV is the commonest cause of recurrent vertigo; it presents as brief spins lasting seconds, triggered by bending down, looking up or rolling over in bed [3, 4]. The elderly might present with falls after getting out of bed [5]. BPV is caused by otoconia dislodged from one of the otolith membranes moving under the influence of gravity, either within a semicircular canal (SCC) duct itself (“canalithiasis”) [6, 7] or while attached to its cupula (“cupulolithiasis”). As the head moves from one position to another with respect to gravity, the otoconia move and increase or decrease the resting activity of canal afferents [8], producing vertigo and a nystagmus with its rotation axis orthogonal to the plane of the stimulated canal [9]. Careful clinical observation and analysis of the exact beating direction (i.e. rotation axis) of this position-provoked nystagmus [10] and of the exact provocative position, allows deductions to be made about which SCC in which ear is being stimulated in which direction—towards the ampulla which is excitatory for the lateral SCC but inhibitory for the vertical SSCs, or away from the ampulla which is inhibitory for the lateral SCC but excitatory for the vertical SCCs. These deductions then guide repositioning manoeuvres. Informative simulations of the presumed movement of the otoconia in the SCCs have been produced [11–15]. A useful collection of BPV videos, uploaded by Dr Dan Gold, can be found on the University of Utah, Neuro-ophthalmology Virtual Education Library (NOVEL) website [16].

## Posterior canal BPV

**Fig. 1:**
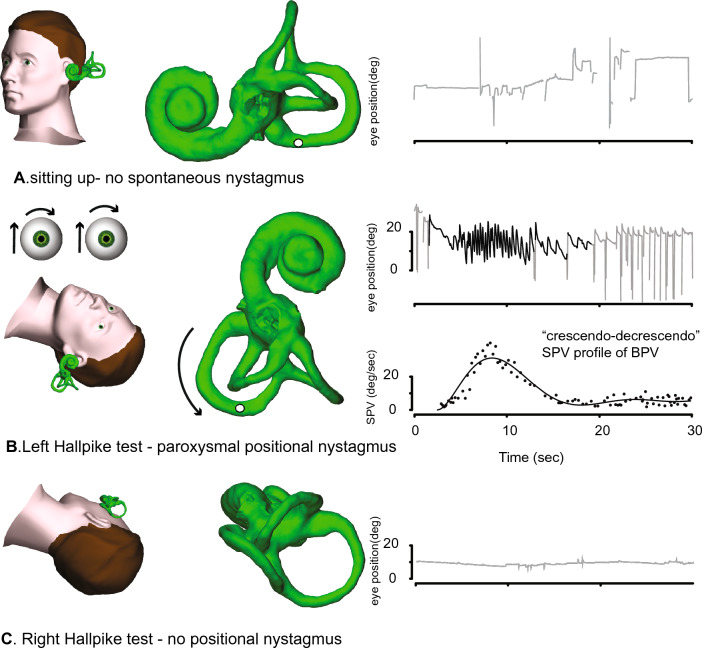
The typical nystagmus profile of right posterior canal BPV. When the subject is upright (**A**), no nystagmus is seen. In the right Hallpike position (**B**), after a latency of 2–3 s, a paroxysm of upbeating torsional geotropic nystagmus is seen, with a crescendo-decrescendo vertical slow phase velocity (SPV) profile

Typical posterior semicircular canal (PSC) BPV accounts for almost 90% of all BPV presentations [17]. The Dix-Hallpike test produces almost immediate geotropic-torsional and upbeating vertical nystagmus indicating that the otoconia are moving in the excitatory direction, that is away from the PSC cupula in the lowermost ear (Fig. 1). (Note: The term “geotropic” when applied to positional nystagmus means that the quick phases of the nystagmus beat towards the lowermost ear; “apogeotropic” means towards the uppermost ear.) Diagnostic criteria for typical posterior canal BPV [18] require: (1) recurrent attacks of positional vertigo or dizziness provoked by lying down or turning over while supine; (2) attack duration of < 1 min; (3) positional nystagmus elicited after a latency of a few seconds by the Dix-Hallpike or the side-lying manoeuvre; (4) geotropic torsional, vertical upbeating (PSC plane) nystagmus lasting < 1 min and (5) that no other disorder better accounts for these findings [3, 4]. Investigations are indicated only when an underlying cause for BPV is suspected [19].

## Horizontal canal BPV

**Fig. 2:**
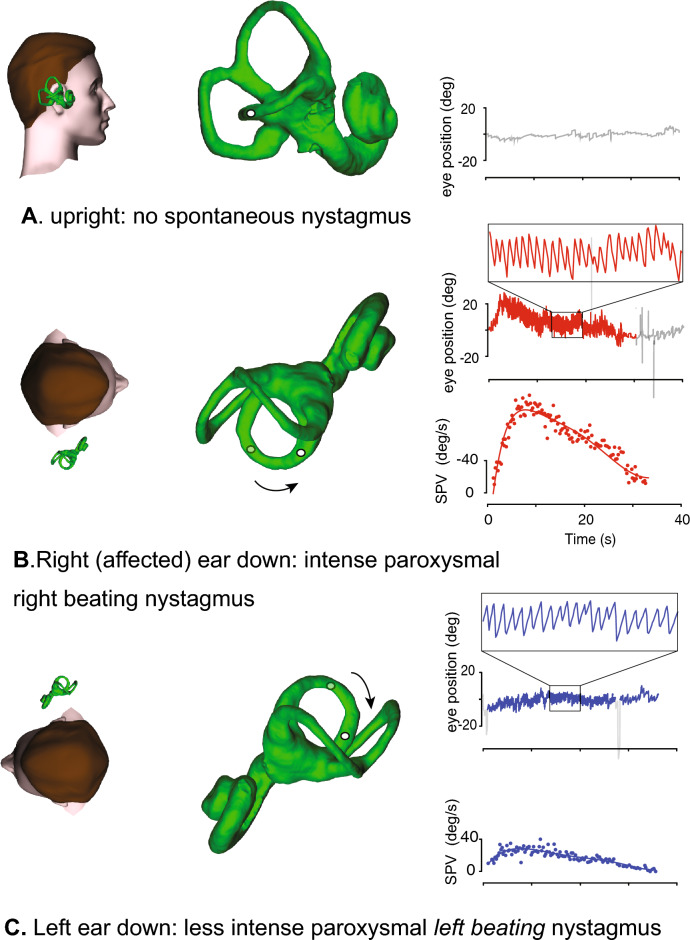
The nystagmus profile of right horizontal semicircular canal BPV. (**A**) When the subject is upright, there is no nystagmus. (**B**) When the affected right ear is placed lowermost, there is after a latency of ~ 1 s or less, a 35s paroxysm of geotropic (right-beating) horizontal nystagmus. It has a crescendo-decrescendo slow-phase velocity profile with a peak velocity of 83 deg/s.  (**C**) When the unaffected left ear is placed lowermost there is a similar duration but less intense paroxysm of geotropic (left-beating) horizontal nystagmus. It also has a crescendo-decrescendo slow-phase velocity profile with a peak velocity of 35 deg/s

Typical horizontal semicircular canal (HSC) BPV—also known as lateral semicircular canal BPV—accounts for about 10% of all BPV presentations. There are several variants, all with some type of horizontal positional nystagmus. Three examples follow. (A) Paroxysmal horizontal nystagmus beating towards the lowermost ear (i.e. geotropic nystagmus). This is attributed to canalithiasis of the HSC in the ear that is lowermost when lying on the side with the higher nystagmus slow-phase velocity (Fig. 2). This nystagmus has a shorter onset latency than PSC-BPV, a crescendo-decrescendo pattern and a relatively longer duration, still less than 1 min [4, 9, 20]. (B) Persistent horizontal nystagmus beating towards the uppermost ear (i.e. apogeotropic nystagmus). This is attributed to cupulolithiasis of the HSC in the ear that is uppermost when lying on the side with the higher nystagmus slow-phase velocity. (C) Persistent horizontal geotropic nystagmus that is symmetrical to each side. This has been attributed to a “light cupula”, i.e. a cupula with a lower than normal specific gravity [21], but not everyone believes this [22]. Both geotropic and apogeotropic horizontal positional nystagmus have also been reported in vestibular migraine [20, 23, 24].

## Treatment of BPV

Typical PSC-BPV can usually be treated effectively and immediately with an Epley or a Semont [25–28] manoeuvre by physiotherapists [29], audiologists [30] or doctors. Some patients learn to treat themselves [31], often by following one of many self-help BPV online videos. HSC-BPV can be harder to treat than PSC-BPV and many different repositioning manoeuvres are used; many are named after the neuro-otologist who first proposed it [28]. The simplest just has the patient lie only on the unaffected side for “as long as possible, preferably all night” [32]. On the basis of modelling, a universal BPV repositioning manoeuvre has been proposed [33]. Unfortunately, even now only a few of the many patients who present to an Emergency Room [34–36] or to a primary care clinic [37] with vertigo even have a Dix-Hallpike test correctly performed; most just have blood tests and brain CT and are prescribed useless anti-emetic tablets.

## Mechanical rotators for treating BPV

There can be practical problems with treating even a simple case of unilateral PSC-BPV. For example, if the patient is 120 kg, 80 years old and has Parkinson's disease, it is impossible to do a proper Epley (or Semont) manoeuvre, or even an accurate Dix-Hallpike test, especially on a narrow examination couch jammed in the office corner up against a wall. Two solutions to this problem are: (1) a home visit: testing and treating the patients in their own home, on their own double bed, with their family helping; with video goggles [38] it is possible to check the nystagmus and to show sceptical family members that there really is something wrong with the patient. (2) Treating the patient in a mechanical repositioning device such as the Epley Omniax rotator (unfortunately no longer made) or the TRV chair, both motor-driven. These devices are suitable and effective [39] for diagnosing and treating patients with BPV that involves multiple canals or those with physical limitations (stroke, spine injuries, morbid obesity) that preclude effective bedside manoeuvres. A transportable manually operated device is also available [40].

## Atypical BPV

There are many other patterns of positional nystagmus in patients who really do have peripheral positional vertigo (i.e. BPV) rather than central positional vertigo [41]. For example, in one type of atypical PSC-BPV the patient has apogeotropic torsional, downbeating nystagmus in the Dix-Hallpike position rather than geotropic, upbeating nystagmus. This could be taken to indicate anterior SCC BPV, but soon the patient develops nystagmus of typical PSC canalithiasis from the opposite side [17, 42]. These patients are thought to have otoconia in the distal part of the non-ampullary arm of the PSC, close to the common crus. Dix-Hallpike testing moves this mass towards the ampulla, thus inhibiting posterior canal afferents and producing an inhibitory torsional downbeating nystagmus. This positional nystagmus can be provoked in either right or left Dix-Hallpike positions, the head-hanging position and sometimes, even in a side lying position; there is a crescendo-decrescendo time-course but no latency and the nystagmus is not completely exhaustible. Rising to the upright position does not reverse nystagmus direction, and it does not fatigue on repeated positioning. Two treatments have been proposed: the second half of the Semont manoeuvre which the patient begins by sitting upright with legs hanging over the edge of the bed, the head rotated towards the healthy ear; then while maintaining this head position, lies onto the unaffected side, thus allowing the otoconia to fall into the common crus and finally the vestibule. The second treatment, termed the “45° forced prolonged position”, requires subjects to lie on the unaffected side with the head turned 45° downwards to bring the non-ampullary arm of the affected posterior canal into a draining position and to maintain this for eight hours [42]. Atypical BPV can be difficult to distinguish from central positional vertigo [43, 44] (see below), and in our view the diagnosis should be made by a neuro-otologist.

## BPV after acute vestibular syndrome

When BPV accompanies or follows an acute vestibular syndrome, the cause of the acute vestibular syndrome should be confirmed with video head impulse testing (vHIT), vestibular evoked myogenic potentials (VEMPs) and audiometry. With BPV secondary to vestibular neuritis [45, 46] there can be impaired ocular VEMPs and horizontal plus anterior canal vHITs but normal cervical VEMPs [47]. In contrast, with BPV after labyrinthitis or labyrinthine infarct, there is also sudden hearing loss [48, 49], and there can be prolonged geotropic or apogeotropic positional nystagmus refractory to treatment (as in cupulolithiasis) and abnormal posterior canal vHIT [50, 51].

## Positional vertigo without positional nystagmus

If the story sounds like BPV, but there is neither positional vertigo, nor positional nystagmus with a correctly done Dix-Hallpike test, it is best to see the patient again [52] rather than order tests. While an unequivocal diagnosis of BPV requires paroxysmal positional nystagmus, some patients who keep having positional vertigo but have no nystagmus during the Dix-Hallpike test can do just as well as those who do have nystagmus after a repositioning manoeuvre [53, 54]. Others only have vertigo after coming up from the Dix-Hallpike test but do have retropulsion and measurable oscillation of the trunk at the same time, possibly due to otoconia on the utricular side of the PSC. These patients can be treated effectively with repeated sit-ups from the Dix-Hallpike position, aimed at liberating otoconia from the short arm of the PSC [55].

## Positional nystagmus without positional vertigo

With removal of visual fixation, an asymptomatic low-velocity (2–5°/s) positional nystagmus, horizontal or vertical of almost every conceivable kind, occurs in many (maybe even most) normal subjects—even in those without a history of BPV or migraine [56, 57]. This needs to be considered when a patient who seems to have had BPV, but is now in remission, has some positional nystagmus in the dark.

## Central positional vertigo and nystagmus

Positional vertigo and positional nystagmus (paroxysmal, persistent or both) can be the presenting feature of some focal lesions and diffuse diseases affecting the cerebellum or the brainstem [58–60]. Downbeating nystagmus on straight head-hanging, upbeating nystagmus on returning to the upright position from supine and apogeotropic nystagmus during the supine head-roll test all occur in central paroxysmal positional nystagmus [58]. The direction of central paroxysmal positional nystagmus aligns with the vector sum of the rotational axes of the semicircular canals that were being inhibited in each position: for example, a straight head-hanging position would inhibit both anterior canals, and so the nystagmus is directly upbeat with no latency to onset and a rapid crescendo phase which decreases exponentially. Time constants for the nystagmus, 3–8 s, correspond to those of the vertical vestibulo-ocular reflex (VOR). The possibility of a central positional vertigo/nystagmus is particularly important to consider in a patient presenting without any other neurological symptoms or signs, just with atypical BPV [17, 41]. Could the cause of the positional vertigo be just migraine [23, 61, 62] or perhaps something more sinister such as a structural lesion? Unfortunately, not even a high-quality, contrast-enhanced MRI with thin, overlapping slices is totally reassuring, as the problem might be an MRI negative, antibody mediated, autoimmune process [63, 64].

## Meniere’s disease

**Fig. 3:**
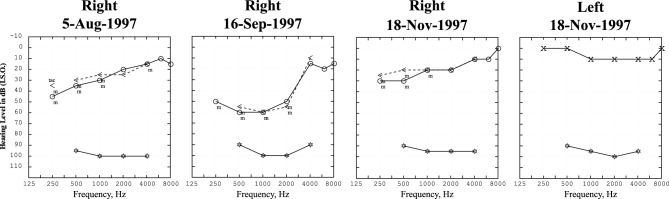
Three sequential pure-tone audiograms from the right ear of a 19-year-old female with Meniere's disease, showing the typical fluctuating, low-frequency, sensorineural hearing loss in the right ear. First audiogram is one month before a vertigo attack, second audiogram is one day after a vertigo attack, and the third two months after the attack. Compare with the normal audiogram from the unaffected left ear. The acoustic reflex thresholds—shown with star (*) symbols at the bottom of each graph—do not change with the increase in subjective pure-tone thresholds in the low frequencies, for example at 1kHz from 30 dB on 5 August to 60 dB on 16 September, indicating recruitment, which is considered characteristic of a cochlear hearing loss. Masked (m) bone conduction thresholds are shown with (<) symbols; there is no conductive component of the hearing loss

So, if it is not BPV, then is it MD or is it VM, or maybe both [65, 66]? There is a close relationship between the two [67], and some actually consider MD to be a vestibulo-cochlear subtype of migraine [68]. The diagnosis of MD is easy if there is unilateral tinnitus and aural fullness with a fluctuating, low-frequency, cochlear-type sensorineural hearing loss which might not be obvious during, or even after, the first few vertigo attacks, but will be eventually [69]. Moreover, the patient might be too dizzy during attacks to notice the hearing problem and could not in any case cooperate with an audiogram. There are smartphone apps offering pure-tone air-conducted audiograms with which it is possible to check if there is a temporary hearing loss with the vertigo attacks [70]. This way any reasonably tech-savvy patient should be able to do their own audiogram on a regular basis in between and just after vertigo attacks. There is no other cause of a low-frequency hearing loss that comes and goes (Fig. 3). Accurate audiological evaluation and interpretation by an audiologist, in cooperation with an otologist, is essential to make the diagnosis of MD. Diagnostic difficulties could arise if the patient has a pre-existing, unrelated hearing loss such as low-frequency conductive (otosclerosis), mid-frequency sensorineural (congenital) or high-frequency sensorineural (age/noise induced), or if the patient has bilateral MD. Drop attacks—in which the patient just drops to the ground—occur in MD as well as in some non-MD aural diseases, but not in migraine [71]. Unfortunately, while most neurologists will order an EEG and ECG in such patients, they will rarely order an audiogram [72]. Repeated attacks of Room Tilt Illusion—suddenly the whole visual world is tilted or even inverted for seconds or minutes—might be a related phenomenon: they can occur in both MD and migraine [73] and perhaps also with TIAs [74] or seizures [75]. Syncope, as a result of the strong vestibular sensation, is rare but potentially dangerous and easy to mistake for a drop attack in a patient with MD [76]. In between MD attacks, there will often be unilateral vestibular impairment of air-conducted ocular and cervical VEMPs and of caloric responses but not of the head impulse test [77–80]. Settings of the subjective visual horizontal (or vertical) might deviate, usually in the same direction as the slow phases of any spontaneous nystagmus [81]. During MD attacks, there is, almost invariably, horizontal nystagmus (sometimes with a vertical component) that can have a horizontal slow phase velocity over 160°/s. The nystagmus first beats towards the affected side (excitatory nystagmus), then towards the normal side (paretic nystagmus) and then again towards the affected side (recovery nystagmus) [82]. Without knowing from hearing loss which is the affected ear, the spontaneous nystagmus direction will not accurately lateralise the MD. This type of nystagmus is enhanced by head-shaking and skull vibration (apparently possible in certain stoical patients) [83]. Rarely, the video head impulse test is temporarily abnormal during an MD attack [84, 85] with either reduced [82, 86] or enhanced [87] responses from the lateral SCC.  It is of interest that the VOR response to pulsed galvanic stimulation can also be enhanced in MD [88].

The vertigo attacks in MD can usually be stopped. Therapeutic total unilateral vestibular deafferentation of the affected ear with vestibular nerve section or labyrinthectomy [89] or partial deafferentation with intratympanic gentamicin [90] can do this, but at the risk of producing imbalance needing long-term vestibular rehabilitation [91–93], especially in the elderly [94]. Intratympanic dexamethasone might be just as good as gentamicin and will not produce imbalance [95]. A low-sodium diet is traditional [96], endolymphatic sac surgery controversial [97, 98], drugs such as betahistine [99, 100] or cinnarizine plus dimenhydrinate [101] still hopeful.

## Vestibular migraine

Many patients with migraine headaches also have balance problems, including vertigo attacks [102–107], and many patients with vertigo attacks or other balance problems also have migraine headaches [61, 108, 109]. There are now official criteria for the diagnosis of VM [105, 110], even though many migraineurs have other, unofficial, balance problems [111] such as chronic subjective dizziness [112], motion sensitivity [113], motion sickness [114], constant rocking sensations (*mal-de-debarquement*) [115, 116], room-tilt illusion [73] or a generalised imbalance [117] which can respond to vestibular rehabilitation [118]. There are some characteristic differences between patients with VM and migraineurs without vestibular symptoms: a longstanding history of migraine with severe headache attacks, aural fullness/tinnitus accompanying attacks, presence of menopause and a history of motion sickness [107, 119]. There might be minor audiologic changes in VM [61, 120] but not a fluctuating, unilateral low-frequency hearing loss as in MD. Children have VM [121, 122]. (They also have BPV [123] but only rarely have MD [124].) Perhaps as a consequence of the vertigo attacks, some VM patients (and also some MD patients) develop psychological problems such as depression [125], anxiety [125–127], panic attacks [128], phobias [129] and of even more concern, possible cognitive impairment [130–135] which might however respond to therapy [136].

**Fig. 4:**
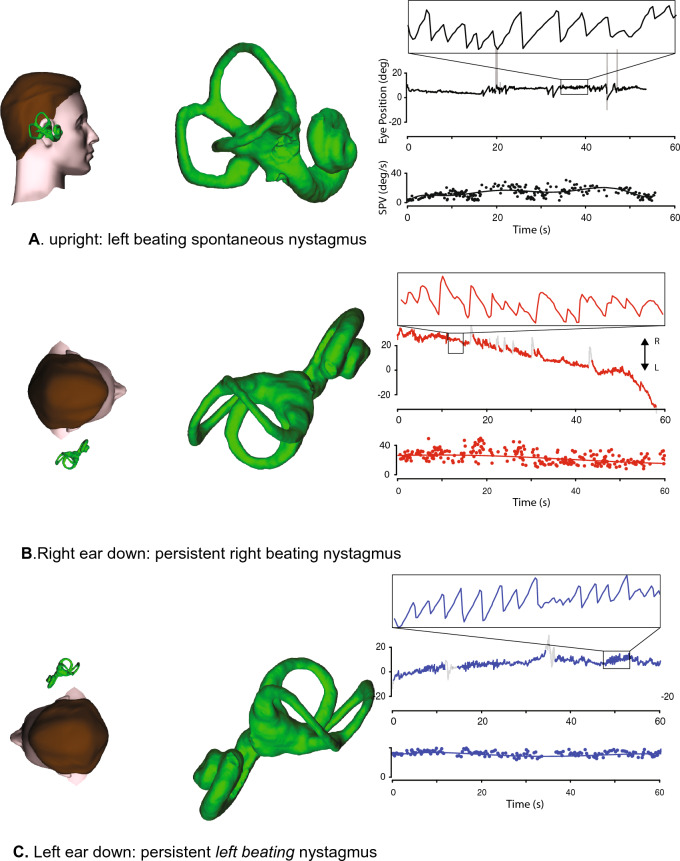
Atypical positional nystagmus in a patient with clinically definite vestibular migraine. Sitting upright (**A**), there is left-beating horizontal spontaneous nystagmus, which persists with the left ear down (**C**), and reverses to right-beating with the right ear down (**B**). With either ear down, there is persistent horizontal geotropic nystagmus, which has a flat slow phase velocity (SPV) profile

Between attacks VM patients can have some low-velocity spontaneous or positional nystagmus in darkness, usually horizontal and around 10°/s or less, but their vestibular function tests (vHIT, caloric and VEMP) are normal [61]. During a VM attack most have a direction-changing or direction-fixed spontaneous nystagmus [137], usually horizontal and less than 15°/s slow phase velocity (but sometimes up to 57°/s), or a persistent positional nystagmus [23] up to 100°/s slow phase velocity in 26% [61] (Fig. 4). Such ictal nystagmus in VM might need to be distinguished from the ictal nystagmus that can occur in MD [78, 82], central vestibulopathy [138] or BPV [139]. When patients have both MD and migraine then things get even more complicated [66, 140–142]. Also, patients can have headache with their BPV [143], and those who have migraine are more likely to have BPV than those who do not [144, 145].

Although there is no solid evidence of measurable benefit from treating or preventing VM [146–149], patients are of course treated [150], usually with drugs that are used for the treatment and prevention of migraine headaches [such as betablockers, pizotifen, tricyclics, anticonvulsants (topiramate, lamotrigine, valproate), cinnarizine, flunarizine or triptans] [109, 151, 152].

## Differentiating MD from VM

In patients with recurrent acute spontaneous vertigo attacks that have been happening for more than say 3 months, MD and VM are the only two realistic diagnoses. If there is also unilateral tinnitus and aural fullness with a low frequency unilateral/asymmetrical sensorineural hearing loss, then it has to be MD. If there are no aural symptoms and no hearing loss, the differential diagnosis will hinge on the vestibular function tests. These should all be normal in VM, but in MD there may be: (1) a canal paresis > 25% on the caloric test with normal lateral SCC vHIT and (2) reduced air-conducted VEMPs, ocular and cervical, on the side with the caloric paresis. The spontaneous nystagmus seen during a vertigo attack is also useful for differentiating MD from VM [153] and is discussed below in our section on vestibular event monitoring.

## Video head impulse testing

Short, fast, head accelerations (head impulses) test SCC afferents in much the same way as patellar tendon taps test 1a afferents. Head impulses test the vestibulo-ocular reflex in response to rapid (2000–3000°/s^2^) head accelerations. The VOR response to these fast stimuli is hard-wired into the neurophysiology of the SCCs and the brainstem; it depends on the resting rate and on–off asymmetry of primary SCC afferents and their robust direct disynaptic or trisynaptic excitatory and cross commissural inhibitory projections via the vestibular nuclei in the pons and medulla to the ocular motor nuclei in the pons and midbrain [154, 155]. The head impulse test [156], specifically the vHIT [157, 158], can detect moderate to severe impairment of any single SCC. It is sometimes (but not always) possible to detect this in the clinical HIT by noting the characteristic compensatory “catch-up” saccades [159–161]. The clinical head impulse test depends, as do other aspects of the neurological examination, on both the clinician’s skill and the patient’s co-operation. If the catch-up saccades have a short latency and so occur while the head is still moving rather than just after it has stopped moving, they will be “covert,” that is, invisible to the clinician but detectible on vHIT [158]. There are now three commercially available vHIT systems: two with goggle-based pupil-tracking cameras and one with a tripod-mounted camera [162]; each system has its strengths and weaknesses and potential pitfalls [163, 164]. With training and practice [165] neurologists, otolaryngologists, audiologists [166] and physiotherapists [167] can all now measure the VOR from each of the six SCCs in almost any reasonably co-operative adult [168] or child [169, 170] in about 20 min. Since 2016 when we wrote the previous version of this review, the yearly number of publications in PubMed dealing specifically with vHIT has increased from 62 to 186. Here, we consider four common clinical situations in which the vHIT could help with diagnosis.

## vHIT during an acute vestibular syndrome

**Fig. 5:**
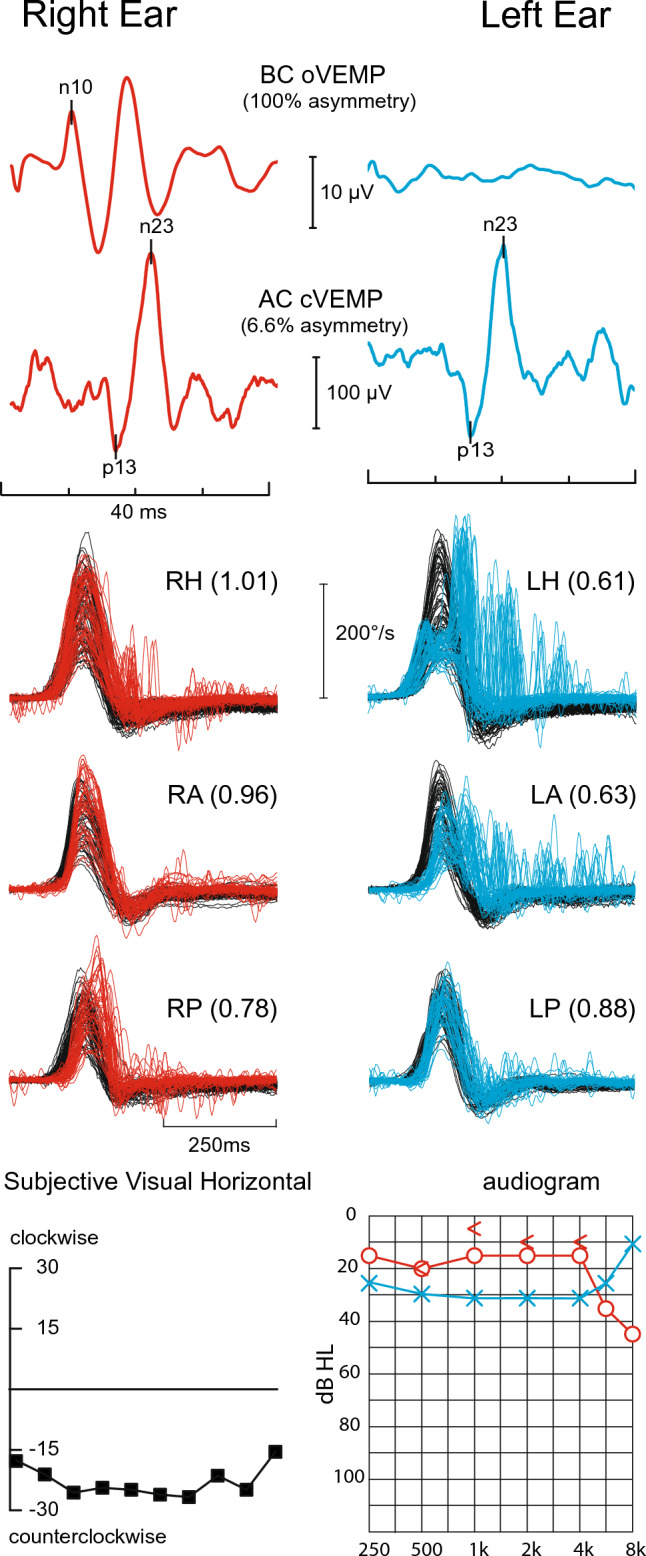
Vestibular neuritis. Vestibular test profile of a patient with left vestibular neuritis who presented with isolated acute spontaneous vertigo lasting three days. The vHIT shows reduced gain from the left horizontal (0.61) and anterior (0.63) semicircular canals with abnormal catch-up saccades. The ocular VEMP, indicating dynamic utricular function, is absent from the left ear, but cervical VEMPs, indicating dynamic saccular function, are symmetrical. cVEMP amplitudes divided by background rectified EMG activation show only a 6.6% asymmetry—abnormal in our laboratory is > 35%. The subjective visual horizontal, which tests the left–right balance of static utricular function, shows a very large (28°) counterclockwise (i.e. towards the left ear) offset indicating reduction in left utricular function. The audiogram shows only a mild, slightly asymmetrical (left > right) high-frequency hearing loss, almost certainly entirely unrelated to the vestibular neuritis

The patient is seen, usually in an Emergency Room, during her first-ever attack of acute, spontaneous, isolated vertigo. Assuming there is no simultaneous acute unilateral hearing loss (neurologists rarely ask about and almost never test for hearing loss), the two main diagnoses are vestibular neuritis and posterior circulation stroke involving the cerebellum and perhaps the brainstem vestibular nuclei. A competent, focused clinical examination which includes a head impulse test, such as *HINTS* [171, 172] or *STANDING* [173] can usually distinguish between the two. Videonystagmography plus vHIT [174–177] will double the rate of correct diagnosis [178]. In acute vestibular neuritis there is sudden unilateral loss of vestibular function [179]. All three SCCs might be involved or only the lateral and anterior which suggests involvement of only the superior vestibular nerve. A patient with left superior vestibular neuritis will have a horizontal/ torsional nystagmus beating to the right, more vigorously in right than in left gaze, suppressed by visual fixation and almost always a clinically obvious impairment of the left horizontal SCC VOR on the bedside HIT [161]. Here vHIT can provide objective, quantitative measures of the VOR from all six SCCs [180], documenting that there really is unilateral impairment of left lateral and anterior SCC function. Vestibular testing can be completed by finding a leftward offset of the subjective visual horizontal (or vertical), loss of left ocular VEMPs indicating impaired utricular function with intact cervical VEMPs [181, 182] indicating preserved saccular function [47, 156, 183] (Fig. 5). Selective inferior vestibular neuritis [157], affecting just the PSC, can only be confidently diagnosed with vHIT and corroborated by finding an absent cervical VEMP (from the saccule) and a preserved ocular VEMP (from the utricle) [184]. In contrast to acute vestibular neuritis, an acute cerebellar/brainstem infarct might not impair the VOR, so the patient will have a normal clinical HIT, a normal or near-normal vHIT [185–187], sometimes not even nystagmus [176], and may just complain of imbalance [188]. Here the logic is counter-intuitive: it is a normal test result, in this case the normal head impulse test (and no nystagmus), that indicates a potentially serious condition. Acute cerebellar infarction is not a diagnosis to miss [189], as there is chance of foramen magnum herniation needing immediate posterior fossa decompression [190] to prevent death or permanent disability [191], whereas an unequivocally abnormal test, the vHIT, indicates a potentially safe-to-discharge condition—vestibular neuritis. Two other conditions that can produce acute, isolated, spontaneous vertigo, MD and VM, also do not show impairment of the VOR on vHIT; they can be hard to differentiate from cerebellar infarction in the acute phase. However, it is unusual in MD for there not to be or have been unilateral tinnitus, fullness, and low-frequency hearing loss, even during the first attack—see above. On the other hand, patients with an MD vertigo attack are usually too busy being dizzy to complain about or even to notice a minor hearing problem, especially in the masking din of an Emergency Room and if nobody asks about it and if nobody can test for it. A severe, first-ever VM attack might be even more difficult to distinguish from cerebellar infarction—even by an experienced neuro-otologist. A combination of one, maybe even two, negative diffusion-weighted MRI scans [192] and a detailed headache history once the patient has recovered is probably the only way. The editor of *Practical Neurology* gives a clear and concise personal account of what it is like to have, and to have had, acute vestibular neuritis [193].

## vHIT after an acute vestibular syndrome

The patient is seen days, weeks—whenever she can get an appointment—after such an attack. She might be asymptomatic and simply wants to know what happened and whether it could happen again. Or she might be complaining of persisting imbalance, because she really did have acute vestibular neuritis and while her brainstem has compensated [194], her peripheral vestibular function has not fully recovered and she now has chronic vestibular insufficiency [195], experiencing head movement oscillopsia and a feeling of imbalance with a positive foam Romberg test [196–198]. Or because she actually had a cerebellar infarct. Alternatively, she could be complaining of further, but less severe, vertigo attacks: if the attacks are spontaneous, she might actually have MD; if the attacks are positional, she might have PSC-BPV as a result of the vestibular neuritis [45, 46].

When a patient still has unilateral impairment of peripheral SCC function according to vHIT, caloric or rotational testing [196, 199–201] some weeks after the acute vestibular syndrome, the diagnosis of vestibular neuritis can be safely made in retrospect. If, however, peripheral vestibular function has largely recovered (with or without corticosteroids [202, 203]), the distinction between recovered peripheral (as opposed to centrally compensated) vestibular function [200, 204] and cerebellar infarction cannot be made clinically and will need MRI. If that too is normal, there is a diagnostic problem. Was this actually an MRI negative cerebellar infarct [192] or a cerebellar TIA [205] rather than a recovered vestibular neuritis or even post-stroke BPV [59]? Could the patient have had a cerebellar embolus from paroxysmal atrial fibrillation [206]? There are more questions than answers.

## vHIT with recurrent vertigo attacks

The patient is seen when well, but complains of recurrent vertigo attacks, either spontaneous or positional. If the attacks really are vertigo, then VM, MD, and BPV are just about the only plausible diagnoses. Rarely, recurrent vertigo is cardiogenic [207]. Unfortunately, most patients who have started to have isolated vertigo attacks from vertebrobasilar TIAs will have a stroke long before their appointment comes around [2]. Recurrent vertigo attacks are the most common vestibular complaint in office practice, but vHIT rarely helps as it is usually normal inter-ictally, even in Meniere’s disease [77, 79, 80]. Nonetheless, it is still worth doing: occasionally BPV is secondary to some inner ear disease [19] and so in that case the vHIT could be abnormal. Posterior SCC vHIT might also be transiently abnormal due to canalithiasis itself [208, 209].

## vHIT in chronic imbalance

There are many possible causes for a complaint of chronic imbalance: some neurological, such as sensory neuropathies, extrapyramidal disorders, orthostatic tremor, or normal pressure hydrocephalus, and others not, such as musculoskeletal disorders or mental health issues. What concerns us here is chronic vestibular insufficiency which can either be due to severe unilateral vestibular impairment [93, 195, 197, 210] or moderate to severe, symmetrical or asymmetrical, bilateral vestibular impairment [211–213]. The patient with chronic vestibular insufficiency might have no obvious symptoms while sitting or lying but feels imbalance as soon as she stands or walks. Despite this there might be little clinically obvious impairment of gait or of stance even with eyes closed and feet together—a negative Romberg test. But if the patient now tries to do a Romberg test on a soft surface, say a foam mat [214], then she will sway and could fall [215] if not caught. This is a positive foam Romberg test [198] which is almost diagnostic of vestibular impairment. Patients with proprioceptive impairment such as those with a hereditary neuropathy such as Charcot-Marie Tooth disease [216, 217] or chronic inflammatory demyelinating polyneuropathy [218] or a ganglionopathy such as CANVAS (cerebellar ataxia neuropathy vestibular areflexia syndrome) [219, 220] already have a positive Romberg test on the firm surface such as the floor but will be worse when standing on foam. Patients with bilateral vestibular impairment might also have difficulties with movement strategies, control of dynamics, orientation in space, and cognitive processing [221, 222]. Such patients will also notice vertical oscillopsia during rapid, passive vertical head-shaking [212] due to impairment of the vertical VOR. They might even volunteer, or at least admit, that they have to stop walking in order to see clearly. Having the examiner shake their head up-and-down will drop their vision by at least three lines on a Snellen chart. Bilateral vestibular impairment needs to be severe to be detectable on caloric or rotational tests, as these tests have large normal ranges. vHIT is the most reliable test to detect bilateral vestibular impairment [211, 213, 223, 224] as it has a tight age-adjusted normal range [225] and is even suitable for detecting age-related vestibular impairment, that is “presbyvestibulopathy” [226–228], also called “presbystasis” [229]. Although mild impairment of just one lateral SCC can be detected by caloric testing, it will not produce imbalance if it is only mild. vHIT is the best test for measuring whether vestibular function is by itself impaired sufficiently to produce imbalance. A possible cause of an isolated severe unilateral vestibular loss presenting with chronic vestibular impairment is an unrecognised previous attack of acute vestibular neuritis [230]; the patient might not have had or might not have noticed vertigo. If there is definitely no history of a previous vertigo attack, then a chronic progressive cause of unilateral vestibular loss such as a vestibular schwannoma (hearing should also be impaired) needs to be excluded [231–233]. The cause of non-syndromic bilateral vestibular impairment without hearing impairment usually remains undiagnosed unless it is bilateral sequential vestibular neuritis [234, 235], gentamicin toxicity [236], Wernicke’s encephalopathy [216] or maybe hereditary spastic paraplegia [237]. If accompanied by hearing impairment then other diagnoses need to be considered: hereditary disorders such as Usher syndrome [238, 239] and also acquired diseases such as superficial siderosis [240] and leptomeningeal carcinomatosis [241]. If there is also cerebellar impairment, as shown by an impaired visually enhanced VOR, then CANVAS [242] needs to be considered. If there is paradoxical enhancement of the VOR on vHIT, as well as of the visually enhanced VOR, then autosomal recessive cerebellar ataxia type 3 (ARCA3) which is due to a mutation in the ANO10 gene needs to be considered [243].

## vHIT: potential practical pitfalls

Although vHIT can be quick and easy to do, it requires training, practice and attention to detail [163, 165, 168, 244]. For example, it is important to interact with the patient throughout testing, continually exhorting her to pay attention to the fixation target (as in visual field testing), not to blink, and not to resist or try to help with the passive head turning. It is important to give head impulse stimuli over the entire magnitude range up to 300°/s peak head velocity. Testing the vertical SCCs requires special attention to eccentric horizontal eye position [245]. The reason it is possible to test the three-dimensional vestibular sensory system with a two-dimensional method (the vHIT) is that when the eyes deviate horizontally so that they align with vertical impulses being delivered directly in a vertical SCC plane, then the VOR is entirely vertical; torsional eye movements, which cannot be detected by the video method, are eliminated. vHIT testing using a head-fixed rather than space-fixed visual target—the suppression Head Impulse (SHIMP) paradigm [246]—can give clearer results in patients with many covert saccades, especially those with only a little residual HSC function.

## vHIT and caloric testing

The caloric has been the mainstay of vestibular testing for over a hundred years [247], and it still has a place in some cases with a normal lateral canal vHIT. It is now proposed that vHIT should be the first test done in a patient with a suspected vestibular problem [248, 249]. If the vHIT is abnormal, then there is no point in doing calorics—they will not give any more diagnostic information. If, however, the vHIT data are clean and truly normal over the entire stimulus magnitude range, then it might be worth asking for calorics [80]. For example, in MD the calorics might be impaired even when the vHIT is normal [79, 80, 166, 250]. One explanation for this discrepancy is that since MD preferentially causes impairment of type II vestibular hair cells [251], it will preferentially impair tonic HSC discharges (responsible for caloric responses) rather than phasic discharges (responsible for impulsive responses). Our alternative explanation, that the caloric impairment is a hydrodynamic effect from the swelling of the endolymphatic compartment abolishing the possibility of thermal convection [77]—the main proposed mechanism of caloric stimulation—is not supported by otopathologic studies [252]. Also, in patients with recovered vestibular neuritis, recovery might be less obvious on caloric testing than on vHIT, which means that a patient seen some time after an acute vestibular syndrome who now has a normal vHIT should have a caloric test—as it might still show a canal paresis [253], indicating that it really was vestibular neuritis rather than a brainstem/cerebellar stroke.

## vHIT and VEMPs

VEMPs can give a semi-quantitative measurement of the function of each of the four otoliths—two utricles and two saccules [183, 184]. VEMPs combined with vHIT make it possible to test each of the 10 vestibular organs individually [254]. VEMPs are about as easy or difficult to do as any other evoked potential test in clinical neurophysiology. There are, however, some important specific technical details to follow in order to record meaningful VEMPs [183, 184]: (a) correct calibration of the air-conducted sound stimulus which needs to be loud enough to be effective but still safe [255, 256]; (b) an effective stimulator for bone-conducted VEMPs, such as a triggered tendon hammer or, for more accuracy, an electro-mechanical vibrator such as a Bruel & Kjaer *minishaker*; and (c) measurement of background rectified sternomastoid muscle EMG activation with cervical VEMP to make the left/right asymmetry ratio more accurate. What then are some clinical situations in which VEMP testing might be useful [257]? Consider the patient who is seen weeks after recovering from an acute vestibular syndrome who has no impairment of vHIT, but has a canal paresis on a caloric test [253]. Here, an absent ocular VEMP from ipsilateral utricle would confirm that the patient has had superior vestibular neuritis [181]. Similarly, if the patient has only an impaired posterior canal vHIT, then an absent cervical VEMP from the ipsilateral saccule could support the diagnosis of a previous inferior vestibular neuritis [47, 258]. VEMPs are particularly useful to help decide if a superior canal dehiscence shown on CT is symptomatic [259]: if the VEMP has a low threshold and a large amplitude, then it probably is [260–262].

## Vestibular event monitoring

**Fig. 6:**
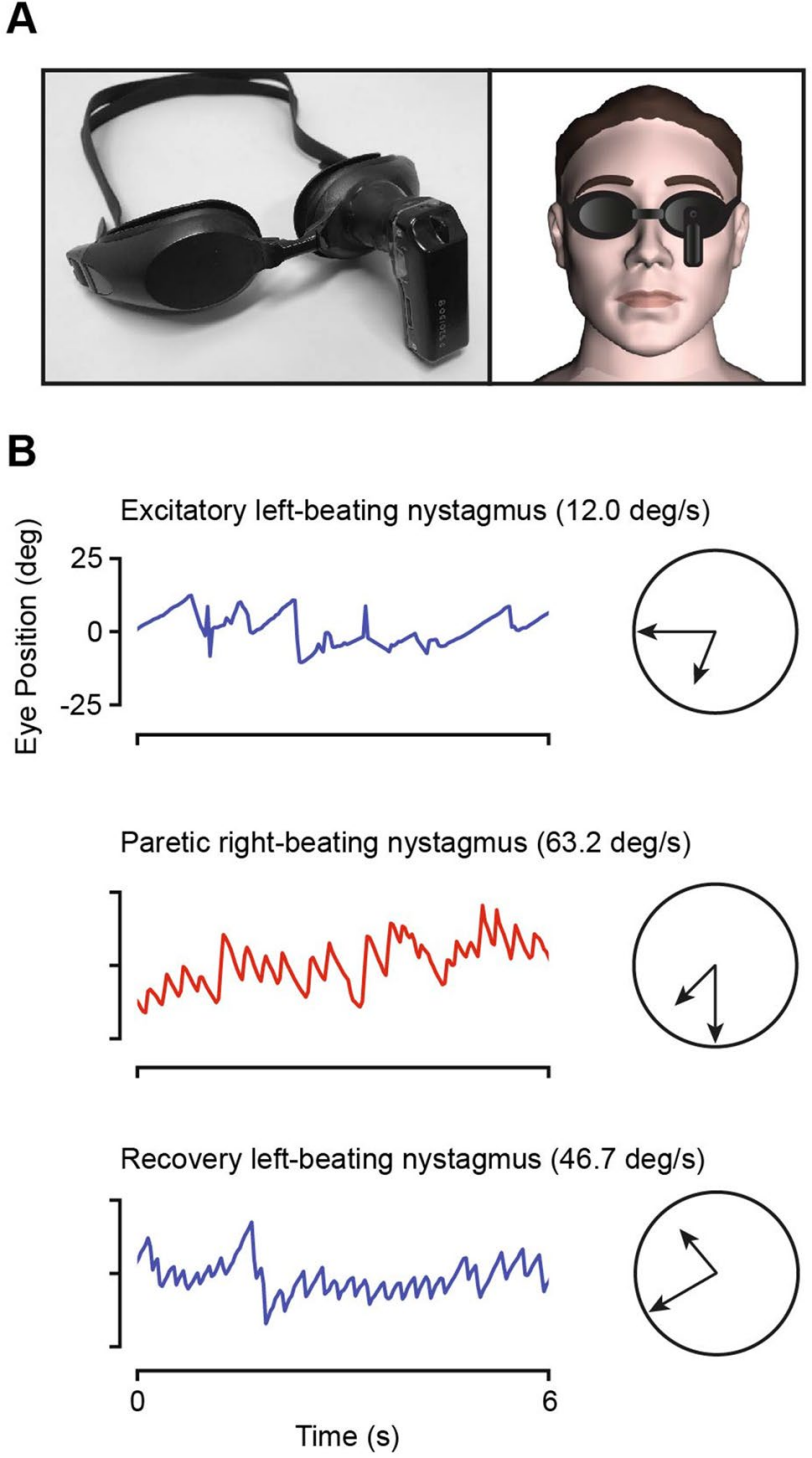
Portable infra-red video goggles used for vestibular event monitoring (**A**), which were used by a patient with left-sided Meniere’s disease to self-capture at home sequential recordings from a single attack. The nystagmus profiles from the recordings (**B**) match the three phases of the classic direction-changing nystagmus seen during an attack of Meniere’s disease: excitatory nystagmus towards the affected side, then paretic nystagmus towards the normal side, and finally recovery nystagmus towards the affected side. Upward deflections indicate rightward eye movements, while downward deflections indicate leftward eye movements

One of the difficulties when diagnosing patients with recurrent vertigo is that they are often asymptomatic when seen in the clinic. Patients with BPV, MD or VM have very mild or no nystagmus between attacks, but will often have marked spontaneous and/or positional nystagmus when symptomatic [61, 82]. This acute nystagmus has diagnostic value. For example, when differentiating MD and VM, spontaneous horizontal nystagmus with slow phase velocity > 12.05°/s during an attack is 82.1% specific for MD, whereas spontaneous vertical nystagmus is 93.0% sensitive for VM [153]. Devices have been developed which allow this nystagmus to be captured during a vertigo episode at home, either by patients self-recording using portable video goggles (Fig. 6) [153, 263] or a wearable electro-oculography device (CAVA) that provides continuous monitoring [264, 265]. Although these devices are not currently widely available—the DizzyDoctor System was marketed but support has recently been discontinued [263]—they will play an important role as a diagnostic aid in the near future. A similar problem applies to patients who are very vertiginous in the Emergency Room, but who are much less symptomatic when reviewed on the ward the next day or in the clinic several weeks later. Using video goggles to record acute nystagmus in the ER helps differentiate between stroke and vestibular neuritis, MD and VM, and BPV and central positional nystagmus [178].

## Machine learning and vestibular diagnosis

In recent years, research into the applications of artificial intelligence and machine learning in healthcare has increased exponentially [266]. The field of neuro-otology has been no exception, and most of the efforts thus far explore the potential for machine learning tools to act as diagnostic decision aids [267]. In general terms, machine learning models take clinical data from patients with conditions of interest and apply various algorithms to ‘learn’ how to distinguish between the diagnoses, without needing to be explicitly programmed with rules to follow. Machine learning methods allow analysis of large, complex datasets including images, and can identify associations that are invisible to the human eye or traditional techniques. Unlike a clinician, their diagnostic performance is never affected by fatigue or carelessness.

The differential diagnosis of vertigo is a problem that is well suited to machine learning as there are often only a few plausible differentials, particularly within a specific vertigo syndrome. Machine learning methods have been applied to clinical data from history [268], examination findings including video eye recordings [269–273], vestibular function tests such as vHIT [274] and VEMPs [275], or a combination of the above [276–278]. Many studies report excellent model performance, including models achieving accuracies and/or area-under-the-curve scores of 0.95 or higher for distinguishing stroke from vestibular neuritis [279], VM or MD from other causes of dizziness [280], or between various subtypes of BPV [272]. However, such promising results come with caveats. Many models use data collected from a single site and have not yet been validated in populations with, for instance, diverse demographics or different laboratory setups. Furthermore, models are often trained using data carefully selected as being typical or free of artefact [272, 274], with exclusion of rarer conditions (such as cupulolithiasis or anterior canal BPV [270, 272]) or patients with unclear diagnoses [268]. Performance in real-world settings can be poorer than expected [281].

The immediate future of machine learning in neuro-otology lies in models which can be used in real-time by clinicians to assist diagnosis. Given the legal and regulatory complexities, it is likely to be some time before clinicians will be replaced by devices which can provide diagnoses autonomously. Emergency physicians, generalists and primary care physicians would be able to access tools that simulate the diagnostic expertise of the expert neuro-otologist and apply this to a much larger population of vertiginous patients. Ideally, models would use only a minimal number of input variables, so as to optimise clinical workflow and reduce computational power requirements, and would not rely heavily on technical expertise or specialised equipment. Promisingly, machine learning algorithms may be able to identify nystagmus even from low resolution images [271], as well as differentiate between common causes of vertigo using only information from a patient questionnaire [268]. Clinicians must familiarise themselves with the limitations of machine learning models and the risks associated with their use [282, 283], as the implementation of these technologies is inevitable.
